# Health of International Migrant Workers During the COVID-19 Pandemic: A Scoping Review

**DOI:** 10.3389/fpubh.2022.816597

**Published:** 2022-02-16

**Authors:** Adriana Oliva-Arocas, Pierina Benavente, Elena Ronda, Esperanza Diaz

**Affiliations:** ^1^Department of Community Nursing, Preventive Medicine and Public Health and History of Science, Faculty of Health Sciences, University of Alicante, Alicante, Spain; ^2^Pandemic Centre, Department of Global Public Health and Primary Care, Faculty of Medicine, University of Bergen, Bergen, Norway; ^3^CIBER for Epidemiology and Public Health, Health Institute Carlos III, Madrid, Spain; ^4^Unit for Migration and Health, Norwegian Public Health Institute, Oslo, Norway

**Keywords:** scoping review, COVID-19, coronavirus, health, migrant workers, international workers

## Abstract

**Background:**

The coronavirus (COVID-19) pandemic and control measures adopted have had a disproportionate impact on workers, with migrants being a group specifically affected but poorly studied. This scoping review aims to describe the evidence published on the impact of the COVID-19 pandemic on the physical and mental health of migrant workers.

**Methods:**

Papers written in English covering physical and mental health among international migrant workers during the COVID-19 pandemic, retrieved from six electronic databases searched on July 31, 2021, were included. A total of 1,096 references were extracted, of which 26 studies were finally included.

**Results:**

Most of the migrant populations studied were born in Asia (16 of 26) and Latin America (8 of 26) and were essential workers (15 of 26). Few studies described the length of stay in the host country (9 of 26), the legal status of the migrant population (6 of 26), or established comparison groups (7 of 26). Ten studies described COVID-19 outbreaks with high infection rates. Fourteen studies evaluated mental health (anxiety, depression, worries, fears, stress, and post-traumatic stress disorder). Three of the 26 studies presented collateral positive effects of the COVID-19 pandemic because of improved hygiene.

**Conclusion:**

There is a limited number of original publications related to the impact of the COVID-19 pandemic on the physical and mental health of migrant workers around the world. These publications mainly focus on migrants born in Asia and Latin America. The physical, long-term impact of the COVID-19 pandemic has, so far, not been evaluated. The positive collateral effects of improving healthcare conditions for migrant workers should also be further investigated.

## Introduction

The COVID-19 pandemic and the measures adopted to prevent and control its spread have had an enormous impact on the global labor force, changing working and employment conditions, developing new ways of working from home and transforming safe working places into potential infection zones. The impact on the working population has been uneven and has widened social gaps (1). It is known that type of work, working conditions, and social security coverage are among the determinants of health (2, 3). As an airborne infection, the SARS-CoV-2 virus enhances the relevance of epidemiological factors in evaluating the impact of disease among workers. In this context, epidemiological factors include occupational exposure to risk of infection, the existence and quality of occupational health services, the efficacy of diagnosing, tracing and following-up of cases and contacts at work, and the possibility of having to comply with isolation and quarantine measures (4–6).

Migration is an independent determinant of health (7). Migrants have been especially hit by the COVID-19 pandemic in terms of disease and economic consequences, and the role of the different factors underlying this over-representation is not yet clear (8, 9). One possible reason is related to migrants as labor force (10). Migrants are over-represented among essential workers in low-skill professions (cleaners, helpers, construction, and industry) and have generally poorer working conditions, are more often temporally and illegally engaged, and suffer higher unemployment rates (11, 12). In Europe, migrant workers constitute 17% of the labor force (13). The pandemic has further worsened migrants' labor situation, with a relatively bigger increase in unemployment rates compared to non-migrants, and by being less often subject to compensation subsidies and social security help (14). As essential workers, migrants who retained their employment during the pandemic have been under-represented in teleworking and are at increased danger of exposure to COVID-19. Migrants, in general, have been less tested than the majority population. Undocumented migrants are especially afraid of being tested and of the consequences of positive test results (15). Additionally, some labor migrant groups rely on their employers' organization of the workplace according to COVID-19 protocol to be able to comply with isolation and quarantine measures (9).

After the first wave of the pandemic from March to July 2020, which was mostly linked to international travel (16), COVID-19 outbreaks were detected in various occupational settings, often within health care, agriculture, services, and construction, where migrants are over-represented (12). This has not only increased the risk of infection for migrants and their families, in some cases these outbreaks have contributed to the stigma and discrimination migrants face, which might have further increased their risk of disease (17). In some places, access to the healthcare system, specifically to occupational health services, is restricted for non-nationals or temporary workers, further increasing the risk of infection (8).

In this scenario, the health of labor migrants has probably deteriorated more than that of other workers during the pandemic because of higher exposure, higher susceptibility, and worse consequences of the disease (18). Although scientific research on COVID-19 pandemic is being published at an astonishingly fast pace, it has been suggested that the health of labor migrants has been neglected (19). To shed light on this particularly vulnerable group of migrants in this review, we describe the evidence published on the impact of the COVID-19 pandemic on the physical and mental health of labor migrants.

## Materials and Methods

### Study Design

This scoping review aims to describe published evidence on the impact of the COVID-19 pandemic on the health (physical and mental) of migrant workers. In our study, we used the methodological approach proposed by Arksey and O'Malley to enable the replication and strengthen methodological rigor (20).

The inclusion criteria for the scoping review were:

1) The studies must be written in English.
2) The studies must cover three topics: health (physical and mental), international migrant workers, and COVID-19.

The exclusion criteria for the scoping review were:

1) Commentaries, protocols, brief reports, perspectives, research letters, letters to the editor, conference abstracts, systematic comparative assessment, narrative and systematic reviews.
2) Studies describing possible risks or stress factors for COVID-19 but not analyzing their impact on health.

### Search Strategy

We selected six electronic databases covering a wide range of disciplines and methodologies: Scopus, Web of Science, CINAHL, Medline, PsycINFO, and Embase. The terms and strings were designed with the help of specialist librarians from the Universities of Bergen and Alicante during July 2021. To define our search terms, we used the PICO search strategy tool (Table 1). Only temporal limits were established on the searches (2019–2021) due to the discovery and global spread of a new coronavirus variant (SARS-Cov-2) in December 2019 in Wuhan (China). Because of the nature of the databases, our search strategies combined Medical Subject Heading (MeSH) and free-text terms. Search terms were truncated to guarantee all relevant articles were included in the analysis. The last review and extraction of information from the corresponding databases was performed on July 31 2021. The results of the electronic search were exported to the Endnote and Mendeley bibliographic managers and duplicates were eliminated.

**Table 1:** PICO search strategy and search terms.

PICO^*^search strategy tool	**Search terms**	
P (participants)	Migrant workers	- (migrant^*^ or migrat^*^ or immigra^*^ or emigra^*^ or foreign) AND (work^*^ or labor or labor or occupation^*^ or employ^*^ or unemploy^*^) - economic NEAR/3 (migrant^*^ or migrat^*^ or immigra^*^ or emigra^*^) - international NEAR/3 (worker^*^ or workforce or employe^*^)
I (intervention/exposure)	COVID-19	- (coronavirus-19 or covid19 or covid-19 or covid2019 or ncov19 or ncov-19 or sars-cov-2 or sars-cov2 or sarscov2 OR sars-coronavirus2) - [(novel or new or noveau) AND (cov or ncov or covid or coronavirus^*^ or “corona virus^*^” or pandemic^*^)] - [covid NEAR/3 (“virus disease^*^” or “virus infection^*^”)] - (covid AND pandemic^*^) - (betacoronavirus^*^ AND pandemic^*^)
O (outcomes)	Health (physical and mental)	- Health

* *(“P” AND “I” AND “O”). C (comparisons) not available*.

### Data Extraction

After the bibliographic search in the selected databases, a total of 2,102 results were obtained (519 in Scopus, 401 in Web of Science, 296 in CINAHL, 360 in Medline, 50 in PsycINFO, and 476 in Embase). After removing duplicates, we ended up with 1,096 papers. Potentially relevant articles were screened by title and abstract by two of the authors (AO, PB), excluding those that did not follow the inclusion criteria. If the abstracts were not informative enough, the articles were selected for further analysis in the next round of review. Differences of opinion with regard to eligibility in the first round were resolved through consensus adjudication between the four authors. A total of 55 papers were selected in the first round. Systematic reviews and narrative reviews were included to assess their bibliographies and we ensured that all the studies listed in those reviews were captured in our database searches.

For selected papers, the full text was independently reviewed by the two authors (AO, PB) and all doubts were discussed by the whole research team. Data was extracted from the documents using an Excel template previously designed to collect and summarize this data. The information registered in the template included: main characteristics of the articles (author name, year of publication, the country where the research was carried out, and journal name), design and data collection technique (e.g., type of article, type of research, and source of primary/secondary data), properties of the sample studied (e.g., sample size, sex, age, country, length of stay, legal status, occupation, and a comparison group if any), objectives, main outcomes, main results, limitations, and conclusions. At the beginning of the full-text review, we carried out a pilot using selected sample articles to test and refine the template and discuss discrepancies with all four researchers. The main question to agree upon was the inclusion of papers focusing on social determinants of health. We agreed to include papers that studied the associations between the COVID-19 pandemic and these determinants and their impact on the health of migrant workers. To guide and structure our results according to the main objective of our scoping review, we classified the included articles by type of outcome: positive (if health status improved or remained the same) or negative (if health status worsened). Negative outcomes include mental (e.g., anxiety, stress, or depression) or physical (e.g., headache or chest pain) conditions that emerged or worsened due to the pandemic and the preventive and control measures adopted. In this regard, reported COVID-19 cases were categorized into physical outcomes. One article reported both types of outcomes and we therefore included it in both groups. In order to facilitate the classification of occupational sectors for the migrant population, the official industry guidelines by the United States Department of Homeland Security (21) and WHO classification (22) were used to identify essential workers and the healthcare workforce, respectively.

**Figure 1:**
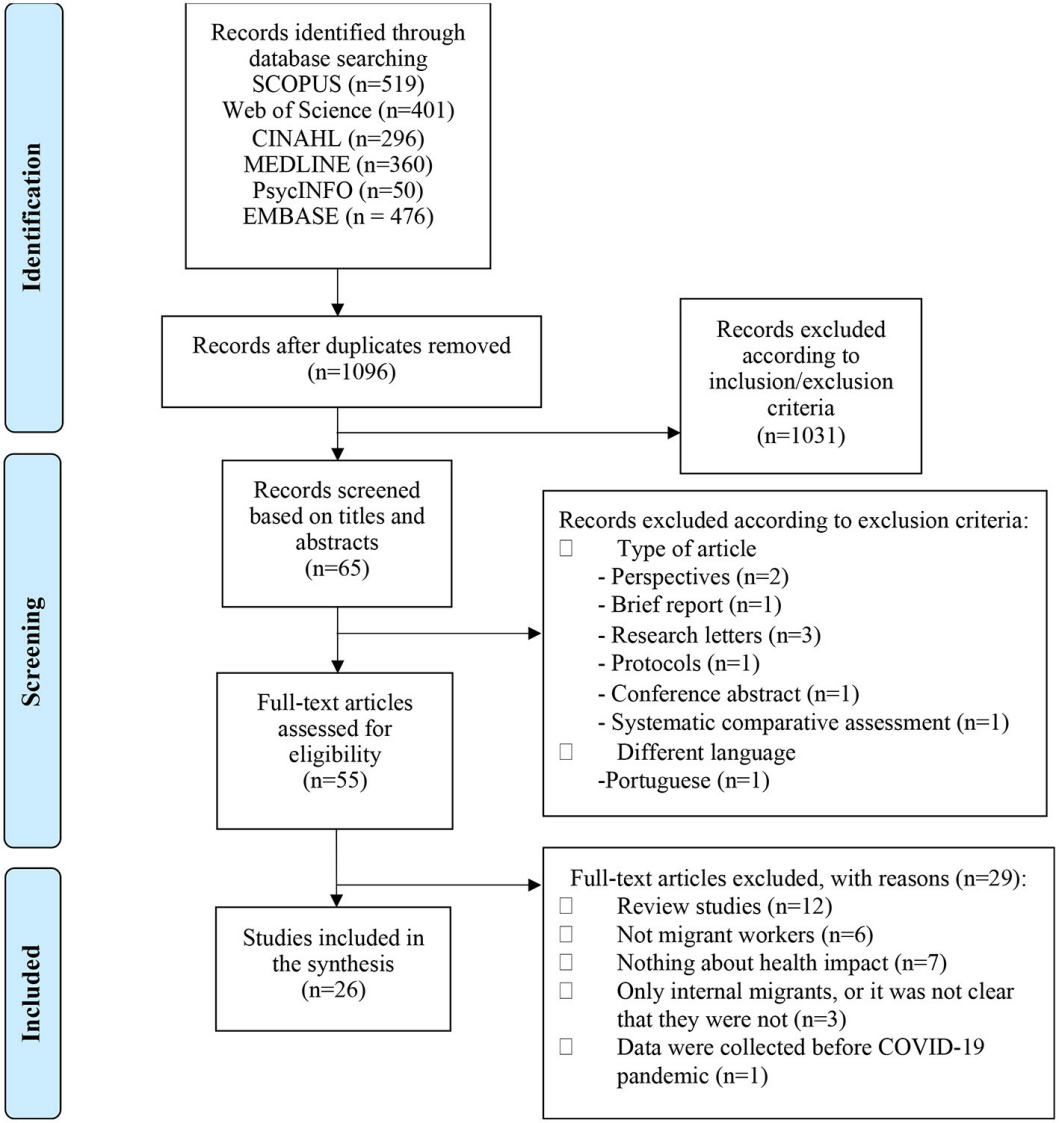
PRISMA flowchart.

A total of 26 papers met the inclusion criteria for this scoping review. Figure 1 shows the screening and selection process followed (23).

## Results

Of the 26 included studies (24–49) almost half (12 of 26) were conducted in Asia (24, 26, 30, 32–35, 37, 41, 44, 47, 49) and 8 in Europe. Fourteen papers used a quantitative design (24, 27, 28, 30, 32, 33, 35, 36, 40, 42–44, 46, 48), 5 applied mixed methods (29, 31, 34, 38, 45) and 23 used primary data sources (24–29, 31, 32, 34, 36–49) (Table 2).

**Table 2:** Description of the 26 studies included.

**Variable**	**Total—*n* (%)**
Total—*N*	26
**Continent of corresponding author**	
Asia	12 (46)
Europe	8 (31)
North America	5 (19)
Oceania	1 (4)
**Data published**	
2020	6 (23)
2021	20 (77)
**Data source**	
Primary	23 (88)
Secondary	3 (12)
**Publication type**	
Original research	24 (92)
Other	2 (8)
**Study design**	
Quantitative	14 (54)
Cross-sectional	11 (79)
Case-control	2 (14)
Case report	1 (7)
Qualitative	7 (27)
Semi-structures interviews	4 (57)
Others	3 (43)
Mixed methods	5 (19)

**Figure 2:**
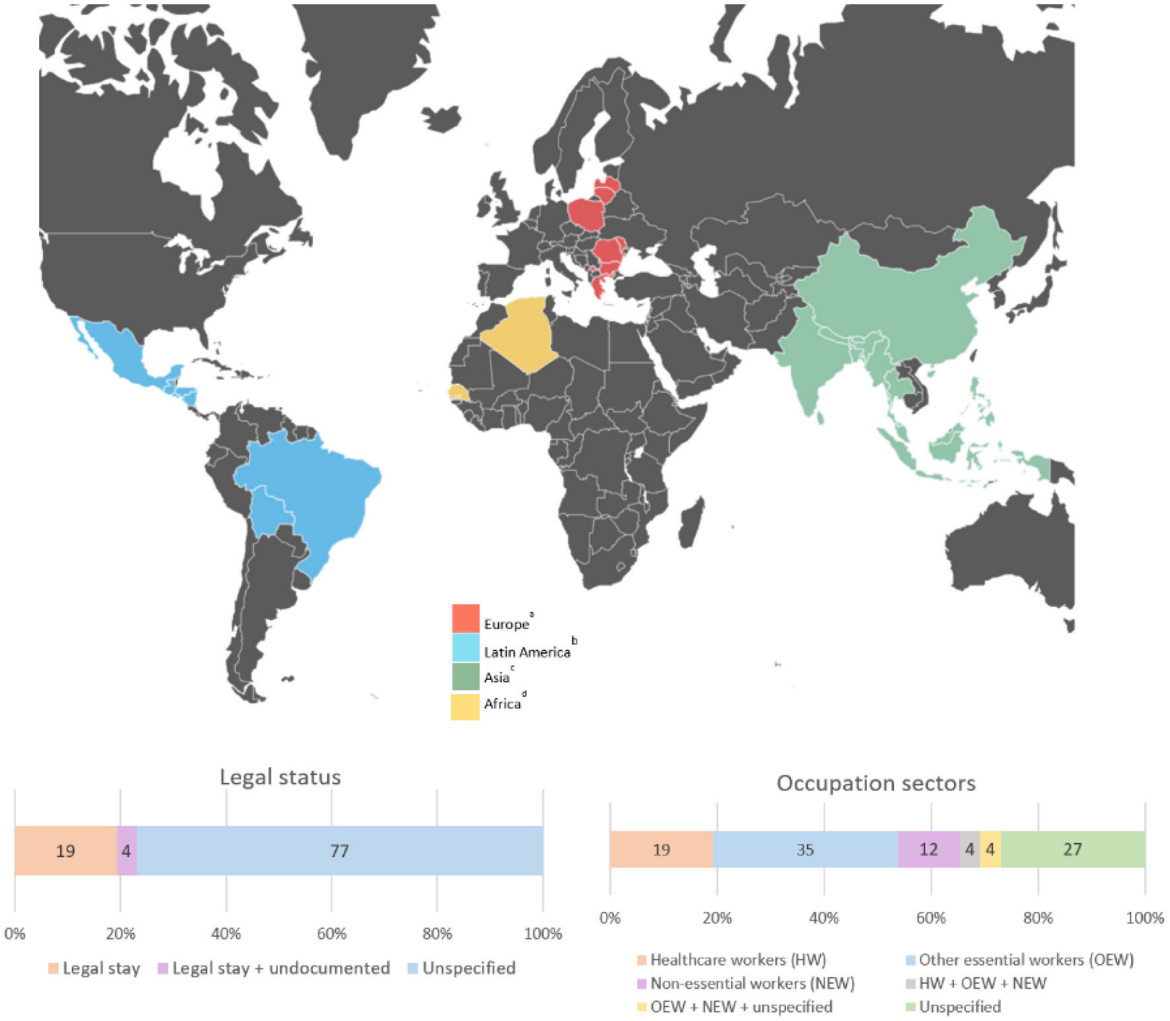
Country of origin, legal status, and occupation sectors of the migrant samples of the 26 studies selected. ^a^Romania, Poland, Greece, Latvia, Moldova, Lithuania, Bulgaria, and Kosovo. ^b^Mexico, El Salvador, Guatemala, Honduras, Nicaragua, Bolivia, and Brazil. ^c^Philipines, India, Sri Lanka, Nepal, Bhutan, Myanmar, Bangladesh, Malaysia, Thailand, China, and Indonesia. ^d^Algeria and Senegal.

Figure 2 shows the geographical, legal, and occupational characteristics of the migrant workers in the 26 studies. Most immigrant populations represented in the studies originated from countries in Asia (16 of 26 studies) (24, 26, 27, 31–34, 37, 38, 41–44, 47–49) and Latin America (8 of 26) (25, 28, 29, 38, 39, 42, 44, 46). Other characteristics of the studied populations were often not described in the methodology of the papers: only 9 studies clearly described migrants' length of stay in the host country (24, 27, 31, 32, 37, 39, 41, 43, 48), most of which were long stays (five or more years) and migrants' legal status was not specified in 20 studies (26–30, 32–34, 36, 37, 39, 40, 42–49). Seven studies had a comparison group (29, 30, 33, 35, 36, 40, 45), most frequently the majority population (6 of 7 studies) (30, 33, 35, 36, 40, 45). Regarding occupation sectors, most of the migrant population were considered essential workers in sectors related to healthcare (6 of 26) (24, 27, 31, 33, 36, 39) and other essential sectors (11 of 26) (25–30, 35, 38, 43, 45, 46) such as agriculture or construction.

As showed in Table 3, most of the selected studies found negative outcomes of the COVID-19 pandemic on the health of migrant workers (24 of 26) (24–29, 31–33, 35–49). Fourteen studies described the impact of COVID-19 on migrants' physical health (25, 27, 28, 33, 35, 36, 39–42, 44–46, 49), 10 studies described COVID-19 outbreaks (25, 27, 28, 33, 35, 36, 40, 42, 45, 46), and the remaining 4 studies mentioned other physical problems such as headaches, sleeping difficulty, respiratory problems, and chest pain (39, 41, 44, 49). Fourteen studies assessed the effects of COVID-19 on mental health outcomes, including anxiety, depression, worry, fear, stress, and post-traumatic stress disorder (24, 26, 29, 31, 32, 37–39, 41, 43, 44, 47–49). Most of these studies linked psychological distress with migrants' economic/financial situation, loss of jobs, food insecurity, exposure to the virus, and/or situation of family members. Anxiety, depression, and stress were related to discrimination and stigma (31, 41, 43, 48, 49).

**Table 3:** Classification of the articles by type of outcomes and their influenced factors.

**Negative outcomes (24 of 26 studies)**		**Positive outcomes (3 of 26 studies)**
**Physical health (14 of 24 studies)**	**Mental health (14 of 24 studies)**	
COVID-19 outbreaks among migrant workers	**Anxiety** *Factor influencing outcome: food insecurity, lack of confidence to care for oneself, poorer general health, finances, job insecurity, increased workload, insufficiency of protective equipment, COVID-19 situation at their home country, lack of trust in NGOs, bad perceived health status and high perceived susceptibility, stigma*	Decreased in dengue transmission *Factor influencing outcome: Quarantine policies*
COVID-19 infection among healthcare workers	**Depression** *Factor influencing outcome: food insecurity, lack of confidence to care for oneself, poorer general health, COVID-19 situation at their home country, stigma*	Good health condition *Factor influencing outcome: Implementation of health protocols, being more conscious regarding eating and living habits*
Healthcare workers infected with COVID-19	**Worry** *Factor influencing outcome: job insecurity, COVID-19 situation at their home country, being exposed to the virus, reading unstructured and unverified news, not being able to support their families at the home country, the impact of homeschooling on children*	
**Headache** *Factors influencing outcome: COVID-19 situation at home country, an increase of work overload*	**Fear** *Factor influencing outcome: job insecurity, not being able to support their families at the home country, working conditions, contracting the disease, transmitting the disease to the person they look after, the health of the family at the home country origin*	
**Worsening** of osteoarticular problems *Factors influencing outcome: change of habits due to lockdown*	**Stress** *Factor influencing outcome: exposure at their workplace or living place, concerns about family wellbeing, finances, language barriers, female gender, job insecurity, bullying and racial discrimination*	
**Loss of appetite** *Factor influencing outcome: psychological distress*	Post-traumatic stress disorder *Factor influencing outcomes: female gender*	
**Sleeping difficulty** *Factor influencing outcome: psychological distress, age, change of habits due to lockdown*		
**Chest pain** *Factor influencing outcome: psychological distress, exposure to chemical products*		
**Respiratory problems** *Factor influencing outcome: exposure to chemical products*		
Allergies *Factors influencing outcome: use of hand sanitizers*		
Weight gain and gastrointestinal disturbance *Factors influencing outcome: change in dietary habits and exercise patterns*		

Three of the 26 studies presented collateral positive effects of the COVID-19 pandemic. One mentioned a decrease in dengue transmission among migrant workers in dormitory sites in Singapore (30), the second mentioned good health conditions for Indonesian migrant workers because of national health protocols being implemented (34), and the third discussed the adoption of new healthy behaviors and the self-perception of being healthier than before the pandemic (41).

## Discussion

This scoping review provides an overview of the published evidence of the impact of the COVID-19 pandemic on the health of migrant workers. It spans the duration of the pandemic until July 2021. We found a small number of original articles focusing on the negative impact of the pandemic on the health of migrant workers, mainly related to COVID-19 outbreaks and the consequences on mental health.

Most of the migrant populations studied during the pandemic worked in occupations defined as essential. Migrant workers are central to the proper functioning of high-income countries and the recent COVID-19 outbreak has only emphasized this fact. Measures to contain the spread of the virus have involved lockdowns and mobility restrictions, while migrant essential workers have had to work in precarious working conditions with a high risk of infection due to limited health and safety protection measures (9). All of this appears to have been detrimental to migrants' mental health, both for those who have remained in destination countries (50) and for those who have been forced to return to their countries of origin (51). According to our results and the research on migrant health in general (52, 53), mental health of migrant workers during COVID-19 seems to be of great interest to researchers. Our research provides evidence on the challenges migrant workers face (e.g., fear of infection, job insecurity, family responsibilities, material and medical needs, and discrimination) and can guide the actions needed to reduce their impact (40, 41, 54).

Of the studies selected in this review, more than half focused on the physical health of migrant workers, but most reported short-term infectious outbreaks. A few articles reported other physical health problems. Among these, respiratory problems, chest pain, and allergies were associated with exposure to chemical products such as hand sanitizer and cleaning products. A limited number of articles also identified sleeping difficulties associated with psychological distress and change in habits due to the lockdown. One study mentioned weight gain and gastrointestinal disturbance because of changes in dietary habits and exercise patterns. Some evidence has been published on isolation, quarantine, and confinement and their effect on physical activity reduction and dietary changes as possible risk factors for the development of chronic diseases in the general population (55–57). All these issues affecting the migrant population require in-depth research.

Most of the studies identified in this scoping review focused on the first wave of the pandemic and therefore do not provide information on the long-term consequences. As is otherwise common in the literature related to the pandemic, research related to COVID-19 and the health of migrant populations has focused on the negative effects of the pandemic and its control and preventive measures. However, for many essential workers, sanitary conditions have improved during the pandemic because of stricter hygiene norms. We found a few studies on the positive impact of COVID-19 pandemic on the health of migrants. Authors like Cornet et al. mentioned the importance of following up on these positive results in the migrant population to sustain the positive changes after the pandemic (58). In addition, Huso et al. suggested the importance of implementing specific government healthcare protocols and quarantine measures that guarantee access to essential services to maintain good health (59).

Most of the studies included in the review had methodological limitations and provided little descriptive information on the migrant samples. Better data related to length of stay, legal status or establishing comparison groups, for example, would have contextualized the results of the studies. Finally, very few studies cover African migrant workers during the pandemic, despite the fact that this migrant group has grown considerably in the last 15 years (60). Although concerns have been raised on the matter, research remains scarce (61).

Table 4 provides a recommendation list for future study based on the research gaps identified in this article.

**Table 4:** Recommendations for future research based on the results of the scoping review.

**Recommendations for future research**
• To explore physical health, other than outbreaks, of migrant workers during the pandemic.
• To study positive health outcomes on migrant workers during the pandemic.
• To evaluate long-term health consequences of the pandemic and its counter-measures on migrant workers.
• To include data such as country of origin, length of stay and legal status to characterize migrant population samples and facilitate comparison across different migrant health studies.
• To examine and compare the effects of different pandemic waves on the health of migrant workers.

Our scoping review has some limitations. Due to the emergence and impact of COVID-19, numerous studies have been published within a short period of time. As a result, we have found high heterogeneity among the studies in terms of design and methodology, which has made it difficult for us to synthesize and compare results. The authors had to make difficult decisions on the inclusion criteria to maintain rigor and achieve an adequate number of selected articles. We decided to exclude gray literature and research published in languages other than English. It is therefore possible that we missed some relevant research work on the subject. However, numerous multidisciplinary databases were consulted to collect as many studies as possible.

## Conclusions

The COVID-19 pandemic has caused widespread economic and social disruption, affecting the lives, livelihoods, and health of people across the globe. The pandemic has also exacerbated existing inequalities among vulnerable groups such as migrant workers. There has been an exponential increase in studies related to the pandemic in general, but the findings of this scoping review indicate that there is a limited number of original publications related to the impact of the COVID-19 pandemic on the health of labor migrants. In particular, the physical long-term impact of the COVID-19 pandemic and the positive collateral effects of improving healthcare conditions for migrant workers should be further investigated. We hope our findings will help focus research attention to important areas that have been overlooked, as well as highlight the need for governments and health institutions to put in place measures to ensure that the health and rights of migrant workers are protected.

## Data Availability Statement

The original contributions presented in the study are included in the article/Supplementary Material, further inquiries can be directed to the corresponding author.

## Author Contributions

AO-A, PB, ER, and ED were involved in all stages of the project and contributed to reviewing the data and writing the manuscript. ER and ED conceptualized the review and provided background information. AO-A and PB designed the search strategy and conducted the screening, data extraction, and synthesis of the literature information. All authors have approved the final manuscript.

## Conflict of Interest

The authors declare that the research was conducted in the absence of any commercial or financial relationships that could be construed as a potential conflict of interest.

## Publisher's Note

All claims expressed in this article are solely those of the authors and do not necessarily represent those of their affiliated organizations, or those of the publisher, the editors and the reviewers. Any product that may be evaluated in this article, or claim that may be made by its manufacturer, is not guaranteed or endorsed by the publisher.
