# Supplementary material for: Health of International Migrant Workers During the COVID-19 Pandemic: A Scoping Review
Source: Front Public Health. 2022 Feb 16;10:816597. doi: 10.3389/fpubh.2022.816597 (PMC8888537; doi:10.3389/fpubh.2022.816597)
Supplement: Supplementary file 1 [file Table_1.DOC]

**SUPPLEMENTARY MATERIAL**

**Supplementary table 1.** Main features and results of the 26 studies included in the scoping review.

| **Title** | **Authors, Country, and Year** | **Type of research and participants** | **Aim/Objectives** | **Main health-related outcomes** | **Main results regarding to health outcomes** |
| --- | --- | --- | --- | --- | --- |
| A rapid assessment of migrant careworkers' psychosocial status during Israel's COVID-19 lockdown. | Attal et al. (1), Israel, 2020. | Quantitative, 307 migrant careworkers. | To assess the psychosocial status and mental wellbeing of migrant careworkers during the ongoing COVID-19 pandemic.  To determine risk factors and protective factors associated with mental distress, anxiety, and depression.  To determine careworker knowledge of the outbreak and necessary precautions to prevent transmission. | Emotional distress (HSCL-10): Anxiety and Depression. | 39% had mental symptoms (28% were anxious, and 38% were symptomatic for depression). Emotional distress associated with household food insecurity, lack of confidence to care for oneself and employer during the pandemic, poorer general health, non-Philippine country of origin, female sex, and inversely associated with age. |
| An Outbreak of COVID-19 Among H-2A Temporary Agricultural Workers. | Lauzardo et al. (2), United States, 2021. | Qualitative, 100 migrant agricultural workers. | To report on a COVID-19 outbreak in a crew of more than 100 H-2A workers in north central Florida to characterize factors that may contribute to the spread of COVID-19 in this essential workforce. | COVID-19 infection. | SARS-CoV-2 was identified in 91 of 100 workers tested. |
| Centering the Margins: The Precarity of Bangladeshi Low-Income Migrant Workers During the Time of COVID-19. | Jamil et al. (3), Southeast Asia and the Middle East countries, 2021. | Qualitative, 97 Facebook pages (10.000-1.4 million subscribers). | To find out the emerging narratives of negotiating disparities and agentic potentials among the Bangladeshi migrants at the margins. | The impact of job insecurities on migrants and their families, living conditions of and abuses on migrants works, negotiations of mental stress by the marginalized migrants and how community supports helps the migrants to survive during the pandemic. | Worker migrants are losing their jobs and are also worried about being able to support their families back in Bangladesh.  Workers have reported severe financial, physical, and mental/psychological distresses. |
| COVID-19 and Immigrant Essential Workers: Bhutanese and Burmese Refugees in the United States. | Zhang et al. (4), United States, 2021. | Quantitative, 218 essential workers. | Describe COVID-19-related risk factors among Bhutanese and Burmese refugees in the United States. | COVID-19 infection (self-reported). | 7% reported infection with COVID-19. The prevalence of COVID-19 was 14% among essential workers and 2% among non-essential workers. Among 33 infected family members 70% were essential workers. Being an essential worker, having family member with COVID-19 infection and being female were associated with the infection. |
| COVID-19 outbreak among temporary foreign workers in British Columbia, March to May 2020. | Mema et al. (5), Canada, 2021. | Quantitative, 63 temporary foreign workers (TFW’s). | To describe the epidemiological investigation and public health response to a COVID-19 outbreak among temporary foreign workers (TFWs) in an agricultural setting in British Columbia. | COVID-19 cases. | 26 COVID-19 cases were identified among the group of TFWs; no cases were identified among local workers. Cases were primarily male (77%). One case required overnight hospitalization for pneumonia. |
| COVID-19 Pandemic Among Immigrant Latinx Farmworker and Non-farmworker Families: A Rural-Urban Comparison of Economic, Educational, Healthcare, and Immigration Concerns. | Quandt et al. (6), United States, 2021. | Mixed, 105 farmworkers and non-farmworkers. | To describe the experiences of mothers of Latin farmworkers families in four domains.  To compare the experience of rural and urban immigrant Latinx families. | 1) Work and household economics.  2) Childcare and education.  3) Healthcare.  4) Community social climate concerning discrimination and racism. | More acute economic effects for urban families. Rural workers reported fewer workplace protective measures for COVID-19. For both groups, fear, and worry, particularly about finances and children, dominated reports of their situations with numerous reports of experiencing stress and anxiety. |
| Decreased dengue transmission in migrant worker populations in Singapore attributable to SARS-CoV-2 quarantine measures. | Lim et al. (7), Singapore, 2021. | Quantitative unspecified. | Identify the potential causal effects of confining migrant workers to dormitory sites on reported dengue cases but considering social distancing and quarantine policies as a natural experiment. | Reported cases of dengue. | A total reduction around 432 reported dengue cases over 10 weeks attributable to quarantine policies (RR reduction 68.5%). In the general working population, a total increase of around 1450 reported dengue cases in the same time period (RR increase 63.5%). |
| Experiences of New Zealand registered nurses of Chinese ethnicity during the COVID-19 pandemic. | Song et al. (8), New Zealand, 2021. | Mixed, 51 migrant nurses. | Examine the experiences and challenges that registered nurses of Chinese ethnicity encountered during the COVID-19 pandemic in New Zealand. | 1) Challenges and concerns.  2) Positive working experiences.  3) Strategies used to manage challenges. | 47% participants reported negative experiences including racial discrimination, workplace bullying and judgement. Chinese nurses reported stress related to nosocomial infection, concerns about family well-being, and received bullying and racial discrimination.  53% participants reported positive working experiences including support received in the workplace and positive recognition by the public in New Zealand. |
| Feeling Anxious amid the COVID-19 Pandemic: Psychosocial Correlates of Anxiety Symptoms among Filipina Domestic Helpers in Hong Kong. | Yeung et al. (9), Hong Kong, 2020. | Quantitative, 295 domestic helpers. | To examine the psychosocial correlates of anxiety symptoms among Filipina domestic helpers (FDHs) in Hong Kong amid the COVID-19 pandemic. | Psychological well-being: anxiety symptoms correlation with working environment, COVID-19 job arrangements, coping resources, COVID-19-specific worries. | The insufficiency of protective equipment, increase workload and worries about being fired if getting COVID-19 were significantly associated with probable anxiety. |
| Healthcare workers in Singapore infected with COVID-19: 23 January-17 April 2020. | Wong et al. (10), Singapore, 2021. | Quantitative, 88 healthcare workers. | Describe the characteristics of health care workers (HCWs) infected with COVID-19 and to examine their sources of exposure. | 1) Characteristics of healthcare workers. (HCWs) infected with COVID-19. 2) Sources of exposure. | Chinese and Indians constituted 42% and 32% (ethnicity) and 43% were foreigners. The majority (64%) was serving at frontlines handling patient-facing duties. About 82% acquired the infection locally, of with 40% did not have a clearly identifiable source of exposure.  Exposure from the family/household was most common (28%) followed by workplace (17%) and social interaction (15%). All HCWs were discharged well with no mortality; three (3%) were admitted to the intensive care unit and required increased care. |
| Indonesia migrant worker’s strategy toward covid-19: Study of migrant’s knowledge and host countries’ policy. | Kusumastuti et al. (11), Unspecified, 2020. | Mixed, 30 migrant workers. | To obtain an overview of the health status of the Indonesian migrant workers, the social dynamics, and the state policy in the place where they live in. | 1) Health status.  2) Strategy of Indonesian migrant workers to deal with COVID-19 in the destination country. | Indonesian migrant workers have implemented the health protocols properly to avoid the spread of Covid-19. Health condition is well maintained during the pandemic in the country they settle in.  The good health condition is also supported with the government policies and health facilities in the country. |
| Migrant workers and COVID-19. | Koh (12), Singapore, 2020 | Quantitative, 17758 low or semiskilled workers. | To examine the number or COVID-19 cases from March to May 2020; the cause of a surge of cases in April 2020; the national response to the huge increase in cases; and regulations on migrant worker accommodation. | 1) COVID-19 cases.  2) National response and regulations. | A marked surge of cases in April 2020 caused by a large number of locally transmitted infection. The majority of cases occurred among an estimated 295 000 low-skilled migrant workers living in foreign worker dormitories. As of 6 May 2020, there were 17 758 confirmed COVID-19 cases among dormitory workers (88% of 20 198 nationally confirmed cases).  Response included mobilizing several government agencies and public volunteers. |
| Seroprevalence of anti-SARS-CoV-2 IgG among healthcare workers of a large university hospital in Milan, Lombardy, Italy: a cross-sectional study. | Lombardi et al. (13), Italy, 2021. | Quantitative, 4055 healthcare workers. | To assess the seroprevalence of anti-SARS-CoV-2 IgG among health careworkers (HCWs) in our university hospital and verify the risk of acquiring the infection according to work area. | 1) Number of anti-SARS-CoV-2 positive serology according working area (COVID-19 cases)  2) Association of cases to selected variables (age, gender, country of origin, body mass index, smoking, symptoms and contact with confirmed cases). | 4055 HCWs were tested and 309 (8%) had a serological positive test. Higher prevalence was observed among foreign-born workers, employees younger than 30 or older than 60 years and among healthcare assistants.  Working as frontline HCWs was not associated with an increased frequency of positive serology (p=0.42). |
| Stressors and coping strategies of migrant workers diagnosed with COVID-19 in Singapore: a qualitative study. | Yee et al. (14), Singapore, 2021. | Qualitative, 27 migrant workers. | To understand migrant worker concerns and coping strategies during the COVID-19 pandemic to address these during the crisis and inform on comprehensive support needed after the crisis. | 1) Migrant workers concerns during the COVID-19.  2) Migrant worker coping during COVID-19 quarantine.  3)Priorities after COVID-19. | Major stressors in the crisis included the inability to continue providing for their families when work is disrupted, their susceptibility to infection in crowded dormitories, the shock of receiving the COVID-19 diagnosis while asymptomatic, as well as the isolating conditions of the quarantine environment.  The workers coped by keeping in contact with their families, accessing healthcare, keeping updated with the news and continuing to practice their faith and religion. They looked forward to a return to normalcy after the crisis with keeping healthy and having access to healthcare as new priorities. |
| The Impact of the Covid-19 Pandemic and the Lockdown on the Health and Living Conditions of Undocumented Migrants and Migrants Undergoing Legal Status Regularization. | Burton-Jeangros et al. (15), Switzerland, 2020. | Mixed, 108 migrant workers. | To describe the impact of the COVID-19 crisis on the health and living circumstances of precarious migrants in Switzerland and to assess whether those undergoing legal status regularization fared better than undocumented migrants. | 1) Self-rated health.  2) Direct health impact of COVID-19.  3) Well-being.  4) Main immediate concerns in the pandemic context.  5) Difficulties related to basic needs. | Migrants showed high prevalence of exposure to COVID-19, poor mental health along with frequent avoidance of health care. Moreover, the loss of working hours and the related income overlapped with frequent food and housing insecurity. |
| COVID-19 and female immigrant caregivers in Spain: Cohabiting during lockdown. | de Diego-Cordero et al. (16), Spain, 2021. | Qualitative, 15 migrant caregivers. | To investigate their feelings and experiences of caregiving during the COVID-19 pandemic lockdown, with specific focus on their emotional and physical well-being regarding two aspects of their lives: the live-in working conditions and the separation from their families. | Emotional and physical wellbeing. | The moral debt accrued by the caregivers with the family who employ them, while worsening the physical and psychological health of many of the caregivers, due to both work overload and fear of the global pandemic. |
| COVID-19 clinical outcomes and nationality: results from a Nationwide registry in Kuwait. | Hamadah et al. (17), United Kingdom, 2020. | Quantitative, 1123 migrant workers. | To explore whether there is a significant difference in health outcomes between non-Kuwaiti and Kuwaiti patients diagnosed with COVID-19. | COVID-19 cases. | The first 1123 COVID-19 positive patients in Kuwait. About 26% were Kuwaitis and 73% were non-Kuwaiti. With adjustments made to age, gender, smoking and selected co-morbidities, non-Kuwaitis had two-fold increase in the odds of death or being admitted to the intensive care unit compared to Kuwaitis. Non-Kuwaitis had also higher odds of acute respiratory distress syndrome (ARDS) and pneumonia. |
| COVID-19: challenges faced by Nepalese migrants living in Japan. | Bhandari et al. (18), Japan, 2021. | Qualitative, 14 Napalese migrant workers. | To identify challenges faced by Nepalese migrants in Japan as a consequence of the COVID-19 pandemic and to discuss their needs to counter these challenges. | 1) Experiencing psychosomatic symptoms.  2) Adoption of new healthy behaviors.  3) Financial hardship.  4) Family concerns.  5) Reflections on discrimination.  6) Reflections of existing support and expectations of support systems. | The specific impact of COVID-19 among Nepalese migrants regarding their unstable employment conditions, perceived lack of social support, possible obligation to send money home, difficulty in accessing services due to the language barrier, and a lack of effective governmental support from Nepal. Pandemic-related adversity has negatively impacted migrants’ mental wellbeing, exacerbating their vulnerability. |
| Cross-sectional study of SARS-CoV2 clinical characteristics in an immigrant population attended in a Hospital Emergency Department in the Catalunya Health Region in Spain. | Yuguero et al, (19), Spain, 2020. | Quantitative, 633 migrant workers. | To assess the characteristics of the patients who attended a Hospital Emergency Department during the first three waves of the coronavirus pandemic. | COVID-19 cases. | They have analyzed 633 immigrant patients who visited the emergency department during the study period. 50.1% patients were women and 78% of all patients came from Africa. The mean age of the patients was 44.1 years. Most patients (72.5%) were discharged to home after evaluation in the emergency department, especially European patients. One-quarter of patients required social resources to be able to comply with quarantine measures, of whom 87% were African. Forty-seven percent of patients became infected at home and 41% in the workplace. |
| Discrimination and Stress Among Asian Refugee Populations During the COVID-19 Pandemic: Evidence from Bhutanese and Burmese Refugees in the USA. | Zhang et al. (20), Switzerland, 2021. | Quantitative, 218 migrants workers. | To measure COVID-19 pandemic-related discrimination and stress among Bhutanese and Burmese refugees in the USA and to identify characteristics associated with these two measures. | Discrimination and Stress. | Among 218 refugees from 23 states, nearly one third of participants reported experiencing at least one type of discrimination, and more than two-thirds experienced at least one type of pandemic-related stress. Having had COVID-19, having a family member with COVID-19, and being an essential worker were associated with discrimination. Discrimination, financial crisis, and female gender were associated with stress. |
| In this together: Psychological wellbeing of foreign workers in the United Arab Emirates during the COVID-19 pandemic. | Barbato et al. (21), United Arab Emirates, 2021. | Quantitative, 319 migrant workers and their families. | To assessing the psychological impact of the COVID-19 pandemic in a sample of foreign workers in the United Arab Emirates (UAE). | Psychological impact. | High rates of post-traumatic stress, depression, anxiety, and insomnia, especially among women, younger individuals, and those with a previous diagnosis of a psychological disorder. Foreign workers’ perceptions of pandemic severity in their home nations (mild, moderate, severe) were positively correlated with their symptom levels of depression, anxiety, and insomnia. |
| A mixed-methods approach to elucidate SARS-CoV-2 transmission routes and clustering in outbreaks in native workers and labour migrants in the fruit and vegetable packaging industry in South Holland, the Netherlands, May to July 2020. | Boogaard et al. (22), The Netherlands, 2020. | Mixed, 46 SARS-CoV-2 cases in migrant and native workers. | To obtain insight into SARS-CoV-2 clustering and transmission routes during outbreaks in the predominantly migrant workforce of the fruit and vegetable packaging industry of South Holland, the Netherlands, May to July 2020. | SARS-CoV-2 cases and the implementation on preventive measures. | They 46 SARS-CoV-2 cases and 4 outbreaks with a proportional representation of labour migrant and native workers in 6 unrelated facilities. Complete viral genome sequences revealed at least 3 clusters of native workers and labour migrants, 2 within and 1 between facilities. On-site inspections found adequate implementation of preventative measures to which both native workers and labour migrants showed suboptimal adherence. Being a labour migrant was associated with living in shared housing, but not with more contacts or different sources. |
| A Mobile Primary Care Clinic Mitigates an Early COVID-19 Outbreak Among Migrant Farmworkers in Iowa | Corwin et al. (23), United States of America, 2021. | Quantitative, 66 migrant farmworkers. | To present a case study of an early COVID-19 outbreaks among migrant farmworkers in Iowa and describes the role that a nimble and responsive mobile federally qualified health center played in the successful mitigation and response to this outbreak. | COVID-19 cases. | The mobile primary care clinic developed a pandemic responsive model to provide successful mitigation of an early COVID-19 outbreak among essential and highly vulnerable migrant farmworkers. |
| Negotiating Mental Health During the COVID-19 Pandemic: Performing Migrant Domestic Work in Contentious Conditions. | Kaur-Gill et al. (24), Singapore, 2021. | Qualitative, 32 migrant domestic workers. | To document mental health narratives by migrant domestic workers during the COVID-19 pandemic, registering how mental health is negotiated amid dissension in the performance of precarious labor. | 1) Performing caregiving in functional and dysfunctional structures.  2) Benevolent structures.  3) Mental health as structural precarities.  4) Structural Contentions as Lived Experience. | The interplays of mental health meanings situated within a structural context of employment and a cultural environment that manifests unequal power relationships and indebtedness. During the Circuit Breaker, domestic workers in already poor employment conditions found themselves at greater precarity and limited agency. Furthermore, the analysis captures the temporalities of mental health meanings articulated within the performance of conducting migrant domestic work in confined working conditions. Precarious migrant journeys include vicious debt cycles, unethical agents, and corrupt employment practices, detailing the scripts of mental health stressors. |
| Prevalence of Depression, Anxiety, and Stress Among Repatriated Indonesian Migrant Workers During the COVID-19 Pandemic. | Harjana et al. (25), Switzerland, 2021. | Quantitative, 335 Indonesian repatriated migrant workers. | To assess the prevalence and associated factors of depression, anxiety, and stress among these populations during the COVID-19 pandemic. | Prevalence of depression, anxiety, and stress. | The prevalence of depression, anxiety, and stress among repatriated Indonesian migrant workers were 10.15%, 9.25%, and 2.39%, respectively. The risk of anxiety and depression was low among those aged 21–30 years old, who had completed a university degree, were married, and had quarantined for 14 days. Conversely, the risk of anxiety and depression was high among those who had bad perceived health status, high perceived susceptibility, and negative stigma perception. |
| "We also deserve help during the pandemic": The effect of the COVID-19 pandemic on foreign domestic workers in Hong Kong. | Lui et al. (26), Hong Kong, 2021. | Qualitative, 15 foreign domestic workers (FDW) and 3 key informants. | To interview affected constituencies, including FDWs, healthcare professionals and other relatively affected people (e.g., those unable to work or travel), to illustrate how different genders and groups were affected by the COVID-19 pandemic according to their position, income, physical safety, distribution of services and access to personal protective equipment. | 1) Dual country experience during pandemic.  2) Changes in work situation.  3) Hong Kong policies and blindspots. | FDWs were concerned both about the pandemic in Hong Kong as well as the effect it was having directly and indirectly on their families and home countries.  The participants reported changes to their current work situation, including the actual work FDWs must perform for employers as well as their employment status.  The FDW community was directly affected by the policies enacted by the Hong Kong government to control the spread of COVID-19, yet many of these policies and decisions were blind to the experiences of FDWs. |

**Supplementary table 2. Characteristics of the migrant workers’ sample for the 26 studies included in the scoping review.**

| **Title** | **Authors, Country, and Year** | **Length of stay** | **Legal status** | **Country of origin** | **Occupation/Sectors** | **Age** |
| --- | --- | --- | --- | --- | --- | --- |
| A rapid assessment of migrant careworkers' psychosocial status during Israel's COVID-19 lockdown. | Attal et al. (1), Israel, 2020. | Nearly 50% in Israel for more than 5 years. | Legal (visa area). | Philippines (58.6%) and Non-Philippines (India, Sri Lanka, Nepal, Romania). | Careworkers | 37 years (median). |
| An Outbreak of COVID-19 Among H-2A Temporary Agricultural Workers. | Lauzardo et al. (2), United States, 2021. | Unspecified | Legal (H-2A visa). | Mexico | Agricultural workers. | 20-29 years (54%) and 30-40 years (38%). |
| Centering the Margins: The Precarity of Bangladeshi Low-Income Migrant Workers During the Time of COVID-19. | Jamil et al. (3), Southeast Asia and the Middle East countries, 2021. | Unspecified | Unspecified | Bangladesh | Low paying jobs (such as construction sites, janitorial, oil fields, etc.). | Unspecified |
| COVID-19 and Immigrant Essential Workers: Bhutanese and Burmese Refugees in the United States. | Zhang et al. (4), United States, 2021. | Mean 10 years | Unspecified | Bhutan (86.2%) and Myanmar (13.8%). | Non-essential and essential workers (food preparation, food serving, home care, healthcare, and sales and related occupations). | 49% less/equal than 30 years. |
| COVID-19 outbreak among temporary foreign workers in British Columbia, March to May 2020. | Mema et al. (5), Canada, 2021. | Unspecified | Unspecified | Latin America | Nursery and garden centre. | Median 41 years (of confirmed cases). |
| COVID-19 Pandemic Among Immigrant Latinx Farmworker and Non-farmworker Families: A Rural-Urban Comparison of Economic, Educational, Healthcare, and Immigration Concerns. | Quandt et al. (6), United States, 2021. | Unspecified | Unspecified | Mexico 80%, El Salvador 7%, Guatemala 3%, Honduras 4%. | Farmworker and non-farmworkers. | 30-39 years (62%) |
| Decreased dengue transmission in migrant worker populations in Singapore attributable to SARS-CoV-2 quarantine measures. | Lim et al. (7), Singapore, 2021. | Unspecified | Unspecified | Unspecified | Construction workers. | 20-65 years |
| Experiences of New Zealand registered nurses of Chinese ethnicity during the COVID-19 pandemic. | Song et al. (8), New Zealand, 2021. | 67% more than 5 years working as nurses in New Zealand. | Legal (working as a registered). | China | Registered nurses. | 30-39 years (57%) |
| Feeling Anxious amid the COVID-19 Pandemic: Psychosocial Correlates of Anxiety Symptoms among Filipina Domestic Helpers in Hong Kong. | Yeung et al. (9), Hong Kong, 2020. | 6.2 years working in Hong Kong (mean). | Unspecified | Philippines | Domestic helpers. | 31-50 years (74%). |
| Healthcare workers in Singapore infected with COVID-19: 23 January-17 April 2020. | Wong et al. (10), Singapore, 2021. | Unspecified | Unspecified | India (19%), Malaysia (10%), Philippines (3%), Bangladesh (6%) Sri Lanka (2%), China (1%) and Germany (1%). | Healthcare workers. | 35 years (median). |
| Indonesia migrant worker’s strategy toward covid-19: Study of migrant’s knowledge and host countries’ policy. | Kusumastuti et al. (11), Unspecified, 2020. | Unspecified | Unspecified | Indonesia | Unspecified | Unspecified |
| Migrant workers and COVID-19. | Koh et al. (12), Singapore, 2020 | Unspecified | Legal (employment pass or work permit). | Unspecified | Workers in construction, manufacturing, marine shipyard, process, or services sector. | Unspecified |
| Seroprevalence of anti-SARS-CoV-2 IgG among healthcare workers of a large university hospital in Milan, Lombardy, Italy: a cross-sectional study. | Lombardi et al. (13), Italy, 2021. | Unspecified | Unspecified | Unspecified | Healthcare workers. | Mean 45 years (of total sample including non-migrants). |
| Stressors and coping strategies of migrant workers diagnosed with COVID-19 in Singapore: a qualitative study. | Yee et al. (14), Singapore, 2021. | 10 years (median). | Unspecified | India (n=12), Bangladesh (n=11), China (n=3) and Myanmar (n=1). | Unspecified | Unspecified |
| The Impact of the Covid-19 Pandemic and the Lockdown on the Health and Living Conditions of Undocumented Migrants and Migrants Undergoing Legal Status Regularization. | Burton-Jeangros et al. (15), Switzerland, 2020. | Unspecified | Regularized | Latin America 63% (including Brazil and Bolivia), Asia 25% (including India), Non-EU Europe 5% (including Kosovo), Africa 6% (Including Algeria and Senegal) | Mostly domestic and construction workers. | 48 years (median). |
| COVID-19 and female immigrant caregivers in Spain: Cohabiting during lockdown. | de Diego-Cordero et al. (16), Spain, 2021. | From 1.5 years to as much as 22 years. | Unspecified | Latin America (almost a half from Nicaragua n=6). | Caregivers | Between 27-62 years. |
| COVID-19 clinical outcomes and nationality: results from a Nationwide registry in Kuwait. | Hamadah et al. (17), United Kingdom, 2020. | Unspecified | Unspecified | Unspecified | Unspecified | Non-Kuwaiti 41.0 years (mean). |
| COVID-19: challenges faced by Nepalese migrants living in Japan. | Bhandari et al. (18), Japan, 2021. | 1-5 years (42.9%), 5-10 years (28.6%), and above 10 years (28.6%). | Visa/Residence status: student (35.7%), dependent (7.1%), working (35.7%), and long-term (21.4%). | Nepal | Unspecified (Employment status only: full-time worker and part-time worker). | 20-40 years (78.6%). |
| Cross-sectional study of SARS-CoV2 clinical characteristics in an immigrant population attended in a Hospital Emergency Department in the Catalunya Health Region in Spain. | Yuguero et al, (19), Spain, 2020. | Unspecified | Unspecified | Europe (n=52), Asia (n=3) and Africa (n= 499). | Unspecified | 44.1 years (mean). |
| Discrimination and Stress Among Asian Refugee Populations During the COVID-19 Pandemic: Evidence from Bhutanese and Burmese Refugees in the USA. | Zhang et al. (20), Switzerland, 2021. | 9.99 years spent in the USA (mean). | Unspecified | Bhutan and Myanmar. | Essential worker (41.74%). | ≤40 years (76.14%). |
| In this together: Psychological wellbeing of foreign workers in the United Arab Emirates during the COVID-19 pandemic. | Barbato et al. (21), United Arab Emirates, 2021. | Unspecified | Unspecified | Europe (69%), Middle East (14%), India (5%), North America (5%), and other (7%). | Unspecified | 40.6 years (mean). |
| A mixed-methods approach to elucidate SARS-CoV-2 transmission routes and clustering in outbreaks in native workers and labour migrants in the fruit and vegetable packaging industry in South Holland, the Netherlands, May to July 2020. | Boogaard et al. (22), The Netherlands, 2020. | Unspecified | Unspecified | Europe (Poland, Greece, Latvia, Moldavia, Rumania, Lituania, Eastern-European, Bulgaria). | Fruit and vegetable packaging workers. | Labour migrants 33 years (mean). |
| A Mobile Primary Care Clinic Mitigates an Early COVID-19 Outbreak Among Migrant Farmworkers in Iowa | Corwin et al. (23), United States of America, 2021. | Unspecified | Unspecified | Latinx and non-Latinx. | Farmworkers | Unspecified |
| Negotiating Mental Health During the COVID-19 Pandemic: Performing Migrant Domestic Work in Contentious Conditions. | Kaur-Gill et al. (24), Singapore, 2021. | Unspecified | Unspecified | Philippine (n=13), Indonesia (n=16), and South Asia (n=3). | Domestic workers. | Unspecified |
| Prevalence of Depression, Anxiety, and Stress Among Repatriated Indonesian Migrant Workers During the COVID-19 Pandemic. | Harjana et al. (25), Switzerland, 2021. | >5 years (44%). | Unspecified | Indonesia | Unspecified | ≤40 years (87.76%). |
| "We also deserve help during the pandemic": The effect of the COVID-19 pandemic on foreign domestic workers in Hong Kong. | Lui et al. (26), Hong Kong, 2021. | Unspecified | Unspecified | Philippines (n=10), Indonesia (n=1), Thailand (n=1), and Sri Lanka (n=3). | Domestic workers. | Unspecified |

**REFERENCES**

1. Attal JH, Lurie I, Neumark Y. A rapid assessment of migrant careworkers’ psychosocial status during Israel’s COVID-19 lockdown. Isr J Health Policy Res. 2020;9(1):61

2. Lauzardo M, Kovacevich N, Dennis A, Myers P, Flocks J, Morris JG. An Outbreak of COVID-19 Among H-2A Temporary Agricultural Workers. Am J Public Health. 2021;111(4):571–3.

3. Jamil R, Dutta U. Centering the Margins: The Precarity of Bangladeshi Low-Income Migrant Workers During the Time of COVID-19. Am Behav Sci. 2021;65(10):1384–405.

4. Zhang M, Gurung A, Anglewicz P, Yun K. COVID-19 and Immigrant Essential Workers: Bhutanese and Burmese Refugees in the United States. Public Health Rep. 2021 Jan;136(1):117–23.

5. Mema S, Frosst G, Hanson K, Yates C, Anderson A, Jacobsen J, et al. COVID-19 outbreak among temporary foreign workers in British Columbia, March to May 2020. Canada Commun Dis Rep. 2021;47(1):5–10.

6. Quandt SA, LaMonto NJ, Mora DC, Talton JW, Laurienti PJ, Arcury TA. COVID-19 Pandemic Among Immigrant Latinx Farmworker and Non-farmworker Families: A Rural–Urban Comparison of Economic, Educational, Healthcare, and Immigration Concerns. New Solut A J Environ Occup Heal Policy. 2021;30(5):30–47.

7. Lim JT, Dickens BL, Ong J, Aik J, Lee VJ, Cook AR, et al. Decreased dengue transmission in migrant worker populations in Singapore attributable to SARS-CoV-2 quarantine measures. J Travel Med. 2021;28(2):1–5.

8. Song J, McDonald C. Experiences of New Zealand registered nurses of Chinese ethnicity during the COVID‐19 pandemic. J Clin Nurs (John Wiley Sons, Inc). 2021;30(5/6):757–64.

9. Yeung NCY, Huang B, Lau CYK, Lau JTF. Feeling anxious amid the covid-19 pandemic: Psychosocial correlates of anxiety symptoms among filipina domestic helpers in hong kong. Int J Environ Res Public Health. 2020;17(21):1–15.

10. Wong LY, Tan AL, Leo Y-S, Lee VJM, Toh MPHS. Healthcare workers in Singapore infected with COVID-19: 23 January-17 April 2020. Influenza Other Respi Viruses. 2021;15(2):218–26.

11. Kusumastuti A, Arawindha U, Harjo IWW. Indonesia migrant worker’s strategy toward covid-19: Study of migrant’s knowledge and host countries’ policy. In Department of Sociology, Universitas Brawijaya, Malang, Indonesia: IEOM Society; 2020.

12. Koh D. Migrant workers and COVID-19. Occup Environ Med. 2020;77(9):634–6.

13. Lombardi A, Mangioni D, Consonni D, Cariani L, Bono P, Cantu AP, et al. Seroprevalence of anti-SARS-CoV-2 IgG among healthcare workers of a large university hospital in Milan, Lombardy, Italy: a cross-sectional study. BMJ Open. 2021;11(2):e047216.

14. Yee K, Peh HP, Tan YP, Teo I, Tan EUT, Paul J, et al. Stressors and coping strategies of migrant workers diagnosed with COVID-19 in Singapore: a qualitative study. BMJ Open. 2021;11(3):e045949.

15. Burton-Jeangros C, Duvoisin A, Consoli L, Fakhoury J, Lachat S, Jackson Y. The Impact of the Covid-19 Pandemic and the Lockdown on the Health and Living Conditions of Undocumented Migrants and Migrants Undergoing Legal Status Regularization. Front public Heal. 2020;8:596887.

16. de Diego-Cordero R, Tarriño-Concejero L, Lato-Molina MÁ, García-Carpintero Muñoz MÁ. COVID-19 and female immigrant caregivers in Spain: Cohabiting during lockdown. Eur J Women’s Stud. 2021.

17. Hamadah H, Alahmad B, Behbehani M, Al-Youha S, Almazeedi S, Al-Haddad M, et al. COVID-19 clinical outcomes and nationality: results from a Nationwide registry in Kuwait. BMC Public Health. 2020;20(1):1–9.

18. Bhandari D, Kotera Y, Ozaki A, Abeysinghe S, Kosaka M, Tanimoto T. COVID-19: challenges faced by Nepalese migrants living in Japan. BMC Public Health. 2021;21(1):1–14.

19. Yuguero O, Calahorra M, Cuevas S, Gimenez N, Hernandez I, Lacasta JD, et al. Cross-sectional study of SARS-CoV2 clinical characteristics in an immigrant population attended in a Hospital Emergency Department in the Catalunya Health Region in Spain. J Migr Heal. 2021;4:100055.

20. Zhang M, Gurung A, Anglewicz P, Baniya K, Mengxi Y. Discrimination and Stress Among Asian Refugee Populations During the COVID-19 Pandemic: Evidence from Bhutanese and Burmese Refugees in the USA. J racial Ethn Heal disparities. 2021;1-9.

21. Barbato M, Thomas J. In this together: Psychological wellbeing of foreign workers in the United Arab Emirates during the COVID-19 pandemic. Int J Psychol. 2021.

22. Boogaard LH, Sikkema RS, van Beek JHGM, Brockhoff HJ, Dalebout E, de Heus B, et al. A mixed-methods approach to elucidate SARS-CoV-2 transmission routes and clustering in outbreaks in native workers and labour migrants in the fruit and vegetable packaging industry in South Holland, the Netherlands, May to July 2020. Int J Infect Dis. 2021;109:24–32.

23. Corwin C, Sinnwell E, Culp K. A Mobile Primary Care Clinic Mitigates an Early COVID-19 Outbreak Among Migrant Farmworkers in Iowa. J Agromedicine. 2021;26(3):346–51.

24. Kaur-Gill S, Qin-Liang Y, Hassan S. Negotiating Mental Health During the COVID-19 Pandemic: Performing Migrant Domestic Work in Contentious Conditions. Am Behav Sci. 2021;65(10):1406–25.

25. Harjana NPA, Januraga PP, Indrayathi PA, Gesesew HA, Ward PR. Prevalence of Depression, Anxiety, and Stress Among Repatriated Indonesian Migrant Workers During the COVID-19 Pandemic. Front Public Heal. 2021;9:1–8.

26. Lui ID, Vandan N, Davies SE, Harman S, Morgan R, Smith J, et al. “We also deserve help during the pandemic”: The effect of the COVID-19 pandemic on foreign domestic workers in Hong Kong. J Migr Heal. 2021;3:100037.
